# Microbial network and its relationship with plants reflect the restoration of grasslands during a five-year period

**DOI:** 10.3389/fmicb.2026.1902699

**Published:** 2026-07-29

**Authors:** Minghui Zhang, Chunnan Fan, Jinping Zheng, Qiang Liu, Fang Yu

**Affiliations:** 1 Forestry College, Beihua University, Jilin, China; 2 Jilin Provincial Laboratory of Forestry and Ecological Environment, Jilin, China

**Keywords:** grassland, microbe-plant interactions, microbial network, restoration process, soil properties

## Abstract

**Introduction:**

The ecological restoration of degraded grassland has been widely studied in terms of plants, soil, and microbes. The microbial network and its relationship with plants are essential indicators of the grassland restoration process. Across restoration statuses, how the microbial network affects plant-microbe interactions and reflects grassland restoration remains unclear.

**Methods:**

We examined changes in soil microbial networks, plant biomass, and soil properties across early, moderate, and late restoration, as well as stable community status, over a 5-year restoration period. We calculated the grassland restoration index (GRI) to evaluate the restoration process and constructed microbial co-occurrence networks to assess changes in microbial communities over 5 years.

**Results:**

Our results showed the dominant species shifting from Kochia sieversiana to Chloris virgata in early restoration status, and the bacterial network complexity fluctuated over the 5 years, indicating a restoration in early restoration status. In moderate and late restoration statuses, the complexity of bacterial networks gradually increased, suggesting the increased recovery potential in the grassland. However, the GRI showed that the moderate and late restoration statuses did not progress to the next successional status over the 5 years. Moreover, the random forest regression model revealed that high soil pH may limit the restoration process by regulating soil microbial networks.

**Conclusion:**

Our study illuminates the signaling roles of soil microbial networks in the restoration process and suggests that moderate and late restoration statuses may take longer to recover to a stable community status. We emphasized that soil pH may be a key constraint in the different restoration statuses, warranting further experimental testing of pH regulation as a restoration intervention.

## Introduction

Human activities and climate change have led to varying degrees of degradation in grassland ecosystems (Gao et al., 2025). Currently, multiple measures (such as fencing and forage culture) are implemented to restore vegetation and soil in degraded grasslands (Cao et al., 2023; Wang et al., 2018). Vegetation restoration focuses on enhancing plant productivity and reestablishing local native vegetation (Jing et al., 2015). Soil recovery emphasizes restructuring of the soil microbial community, which is more challenging and determines the process of grassland restoration succession (Liu et al., 2019). In recent years, many studies have found that some grasslands remain in a certain restoration status and are difficult to return to their non-degraded status (Kardol and Wardle, 2010). The theory of multiple stable states suggests that grasslands may have natural restoration thresholds due to their soil environments (Liu M. et al., 2025; Chaparro-Pedraza, 2024). These thresholds affect plant growth by regulating the microbial community, thus constraining the ecological restoration process (Liu M. et al., 2025). Therefore, the changes in soil microbial community structure are the key to understanding the process of grassland restoration succession. However, the importance of microbial community dynamics on grassland restoration has only recently been recognized.

Grassland restoration refers to the recovery of the soil environment and plant community through human interference or natural restoration (Bardgett et al., 2021). The ultimate goal of grassland restoration is to restore plants and soil in degraded grasslands to non-degraded conditions (Sun et al., 2020; Feng et al., 2010). Therefore, based on the characteristics of plants and soil, grassland restoration can be classified into early, moderate, and late restoration status (Liu et al., 2021; Liu M. et al., 2025; Pellant et al., 2005). Generally, the vegetation and soil environment in the late restoration status are close to the non-degraded status (Su and Zhang, 2004). Moderate restoration status has high plant diversity, which is the boundary for successful natural recovery (Liu et al., 2021). Early restoration status is difficult to self-recover due to the severe damage to plant communities and soil environments (Dong et al., 2011). For example, fencing and grazing prohibition effectively restore plant communities in the moderate- and late-successional stages of grassland restoration (Sun et al., 2020). In the early restoration status, grassland requires forage cultivation and soil improvement to enhance recovery efficiency (Feng et al., 2010). However, previous studies have shown that plant communities during the restoration process may be difficult to recover to the next restoration status (Hu et al., 2016; Li et al., 2023). This phenomenon may be due to the high soil heterogeneity in grasslands, which leads to an uneven distribution of soil nutrients (Gao et al., 2019). Subsequently, nutrient-rich areas form patches of different plant communities, while some areas remain in their restoration status (Jiang et al., 2010). Moreover, soil heterogeneity is often accompanied by high soil pH and salinization, which aggravated grassland fragmentation and delayed the restoration process (Liu et al., 2021). Therefore, the processes and effectiveness of degraded grassland restoration under different soil environments remain highly contentious.

Soil microbial communities play a crucial role in various ecological processes such as nutrient cycling and plant community succession (Breidenbach et al., 2022). Due to the sensitivity of soil microbes to environmental and vegetation changes, the microbial community structure typically shifts during grassland restoration succession (Li et al., 2016; Akiyama and Kawamura, 2007). Therefore, the dynamics of microbial communities are important factors reflecting the restoration succession process (Fry et al., 2017). In recent years, many studies have examined microbial community dynamics using the microbial network (Guseva et al., 2022; Ding et al., 2023). The complexity of microbial networks explains how microbial communities respond to biotic and abiotic factors through the interrelationships among microorganisms (Xue et al., 2022). Accordingly, the microbial network complexity may change during grassland restoration processes (Ding et al., 2023; Morrien et al., 2017). Generally, increasing microbial network complexity indicates improved soil multifunctionality, suggesting recovery potential during grassland restoration (Chen et al., 2022; Du et al., 2025; Mi et al., 2026). However, microbial networks are highly correlated with the structure of vegetation communities (Finck et al., 2026). Grassland restoration successions are accompanied by transitions in plant community composition and soil environments, and thus may lead to corresponding changes in microbial networks (Jayaramaiah et al., 2026; Song et al., 2025). For example, the plant diversity in the moderate restoration status is generally higher than that in the late restoration status (Liu et al., 2021), which may result in a more complex microbial network in the moderate restoration status. Therefore, the mechanism by which microbial network complexity changes across different restoration statuses remains unclear.

Soil microbes drive the succession of grassland restoration via microbe-plant interactions (Lozano et al., 2014). Positive microbe-plant interactions promote succession under severe soil conditions (Pii et al., 2015). As soil environments and nutrient availability increase, these positive interactions may enhance the competitive advantage of dominant species and slow the succession process (Guo et al., 2019). For instance, low soil nutrient levels in early restoration status inhibit plant growth, while positive microbe-plant interactions can improve plant nutrient absorption efficiency and grassland restoration (Brzostek et al., 2014; Guo et al., 2019). During grassland succession, plants amplify their competitive advantage through positive interactions with microbes, thereby alleviating the transition of dominant species and inhibiting succession (Li et al., 2019). However, the multiple stable states theory suggests that soil conditions across different restoration statuses affect microbe-plant interactions, leading to varying efficiencies in ecological restoration (Liu M. et al., 2025). For example, the light-degraded grassland can rapidly recover to its pre-degraded status (Dong et al., 2020). This phenomenon is due to minimal damage to plants and soil, allowing vegetation to quickly establish positive interactions with soil microorganisms and thereby rapidly return to a non-degraded status (Sun et al., 2020; Gann et al., 2019). In contrast, the early restoration status of severely degraded grassland may last for a long period. Although reseeding forage can restore plant biomass, severe soil environments reduce the establishment of microbe-plant interactions (Dong et al., 2011; Zhang et al., 2021). Overall, there are many debates about the succession mechanisms of degraded grassland restoration. Likewise, regardless of the theory, clarifying the dynamics of microbe-plant interactions is key to revealing the process and direction of grassland restoration succession.

This study aims to determine how microbial community structure and networks affect restoration succession across different restoration statuses. During the 5-year natural recovery period, we conducted three field investigations to assess plant characteristics, soil properties, and microbial communities across varying restoration statuses (i.e., early, moderate, and late restoration, and stable community). Based on previous studies, microbial network complexity increases with the amelioration of soil conditions and the recovery of plant communities (Mi et al., 2026). Meanwhile, high soil pH is a typical feature of degraded grasslands in this area (Liu et al., 2021), and ecological restoration can enhance plant–microbe interactions (Lozano et al., 2014; Kardol and Wardle, 2010). Therefore, we hypothesized that (1) the microbial network complexity increases with the restoration time, which leads to the increasing potential of grassland recovery; (2) microbial networks regulate the restoration process via microbe-plant interactions, and high soil pH may be a key constraint for the grassland restoration.

## Materials and methods

### Sample area description

This study was concluded at the Tongyu Semi-Arid Climate-Environment Field Station (122°52′E, 44°25′N), located in the Songnen Plain, northeastern China. The Tongyu station covers an area of 47.7 hm^2^ and is classified as a meadow steppe, and the dominant species is *Leymus chinensis*. The mean annual precipitation is 404 mm, with 80% falling from May to September. The mean annual temperature is 5.7 °C, with the lowest at −32 °C in December and the highest at 39 °C in June. The soil exhibits strong heterogeneity, including sandy, slightly chernozem, salty, alkaline, and meadow soils (Gao et al., 2019; Liu et al., 2021).

Since 2006, the meadow steppe of this area has been fenced to prevent grazing. This area experienced varying grazing intensities, resulting in grassland degradation before fencing was established (Wang and Ripley, 1997; Jiang et al., 2010). As a result, grasslands in this area vary in their degradation status due to different grazing intensities (i.e., heavy grazing around water sources). From 2006 to 2021, some degraded sites were gradually recovered. However, local grasslands exhibit low soil nutrient levels and high soil salinization, which inhibit the restoration of degraded grasslands (Liu et al., 2021; Zhang et al., 2024). These reasons lead many restored sites to maintain their restoration status. Accordingly, this area can be classified into early, moderate, late restoration status, and stable community status (i.e., stable *L. chinensis* community) according to previous studies and the guideline for grassland degradation and restoration assessment (Su and Zhang, 2004; Pellant et al., 2005). Moreover, we calculated the grassland restoration index (GRI) to confirm the restoration level of each site using the following equation:


$$\text{GRI}=\frac{1}{3}\,\left(\frac{1}{3}\,{\text{P}}_{1}-\frac{1}{3}\,{\text{P}}_{2}-\frac{1}{3}\,P_{3}\right)+\frac{1}{3}\,\left(\frac{1}{3}\,\text{TC}+\frac{1}{3}\,\mathrm{TN}+\frac{1}{3}\,\text{TP}\right)+\frac{1}{3}\,\left(\frac{1}{2}\,\text{SW}-\frac{1}{2}\,\text{pH}\right)$$

where P_1_, P_2_, and P_3_ are the relative abundances of climax species, annual species, and salinization indicator species, respectively; TC, TN, and TP represent the relative concentration of soil total C, N, and phosphorus (P), respectively; SW and pH are the relative soil gravimetric water content and soil pH, respectively. The relative number was calculated by dividing each factor’s value by the sum of that factor across the four successional statuses. The GRI has been proven to determine the different restoration statuses of the meadow steppe in the Songnen Plain (Zeng et al., 2018; Dong et al., 2020; Yang et al., 2021). We treat the GRI number in the stable successional status as 100% of the restoration level. GRI values in the ranges <55%, 55%–80%, and 80%–90% represent early, moderate, and late restoration statuses, respectively. These thresholds are based on a previous study near Tongyu station (Yang et al., 2021), and our field survey verified their accuracy (Table 1).

**TABLE 1:** The aboveground biomass of main plants in the different restoration statuses (mean ± SE, *n* = 4).

Restoration status	Main plant species	Aboveground biomass (g m^–2^)		
		2021	2023	2025
Early restoration status	*Chloris virgata*	61.36 ± 12.26	156.22 ± 7.83	167.40 ± 14.33
	*Kochia sieversiana*	69.74 ± 3.17	47.61 ± 14.41	48.66 ± 4.64
	*Artemisia scoparia*	17.28 ± 1.78	15.71 ± 3.87	14.26 ± 7.64
	Total biomass	148.37 ± 13.75 b	219.54 ± 16.11 a	230.32 ± 20.97 a
	GRI	−0.15 (−98%)	−0.14 (−93%)	−0.13 (−88%)
Moderate restoration status	*Leymus chinensis*	188.90 ± 21.97	206.19 ± 10.19	181.25 ± 26.94
	*Calamagrostis macrolepis*	8.00 ± 1.94	5.26 ± 4.37	17.50 ± 1.97
	*C. virgata*	–	8.32 ± 2.67	11.85 ± 4.41
	*Carex duriuscula*	5.13 ± 0.00	21.18 ± 3.82	9.70 ± 1.42
	*Lespedeza daurica*	10.82 ± 3.80	10.65 ± 2.67	12.97 ± 2.34
	*K. sieversiana*	–	10.77 ± 4.86	10.36 ± 3.44
	*A. scoparia*	6.48 ± 2.04	4.44 ± 2.80	7.17 ± 0.00
	Total biomass	217.43 ± 23.73 a	247.34 ± 22.54 a	232.26 ± 30.16 a
	GRI	0.09 (61%)	0.09 (58%)	0.08 (59%)
Late restoration status	*L. chinensis*	245.07 ± 28.37	247.22 ± 13.73	221.24 ± 33.84
	*C. macrolepis*	58.45 ± 13.76	67.88 ± 24.04	40.32 ± 1.08
	*L. daurica*	68.30 ± 6.53	50.27 ± 5.47	60.81 ± 8.09
	*A. scoparia*	3.13 ± 0.33	2.09 ± 0.00	1.37 ± 0.00
	Total biomass	346.68 ± 63.28 a	335.76 ± 37.96 a	316.91 ± 42.33 a
	GRI	0.13 (83%)	0.13 (85%)	0.12 (85%)
Stable community status	*L. chinensis*	334.17 ± 32.02	344.64 ± 28.00	322.48 ± 13.98
	*C. duriuscula*	20.31 ± 6.31	24.89 ± 12.96	24.12 ± 9.63
	*A. scoparia*	5.17 ± 1.71	4.08 ± 1.03	4.33 ± 1.90
	Total biomass	359.65 ± 28.35 a	372.59 ± 36.54 a	350.78 ± 16.50 a
	GRI	0.15 (100%)	0.15 (100%)	0.14 (100%)

Different letters indicate a significant difference (*P* < 0.05) in different years; – indicates the plant was not detected.

### Field vegetation survey and soil collection

In August 2021, four 10 m × 10 m plots were randomly selected in each restoration status (ND, LD, MD, and SD). To reduce environmental differences, each selected 10 m × 10 m plot contains the four restoration statuses. These plots had at least 200 m to ensure the representativeness of the soil sample. In these plots, each restoration status randomly selected a 1 m × 1 m quadrat for plant and soil collection. Based on previous studies, these sampling sites are reasonable for reflecting the characteristics of the sampling area (Zeng et al., 2018; Liu et al., 2021). In each quadrat, we harvested all plants and measured the aboveground biomass of each plant species. The method for plant collection and measurement is based on a previous study at the Tongyu station (Zhang et al., 2024). In August 2023 and 2025, we randomly selected a 1 m × 1 m quadrat near the 2021 quadrat, respectively. The sampling methods for plants and soil are the same as those in 2021.

Subsequently, we collected the soil sample at the vegetation survey site. After removing the litter, 1 kg of fresh soil was collected from 0 to 15 cm of topsoil using a soil auger (4 cm diameter). Each quadrat was randomly sampled 4 times and combined into a single soil sample for each plot (4 kg). These soils were passed through a 2 mm sieve and divided into two subsamples. One subsample was air-dried to analyze the soil properties (i.e., the C, N, and P contents, soil pH, and gravimetric water content). Another subsample of these soils was immediately stored in liquid nitrogen and transferred to the laboratory for analysis of bacterial and fungal communities.

### Plant and soil sample measurements

Each plant sample was separated and dried at 105 °C for 15 min, then at 65 °C for 48 h to measure biomass. Soil bacterial and fungal communities were measured using high-throughput sequencing. Soil DNA was extracted using the MoBio PowersoilTM DNA Isolation Kits (MoBio Laboratories, Inc., USA). The F: 5′-ACTCCTACGGGAGGCAGCA-3′, R: 5′-GGACTACHVGGGTWTCTAAT-3′ was used to amplify the V3–V4 regions of the bacterial rRNA. The F: 5′-CTTGGTCATTTAGAGGAAGTAA-3′, R: 5′-GCTGCGTTCTTCATCGATGC -3′ was used to amplify the ITS1 primer pairs, regions of the fungal rRNA. Amplicon paired-end sequencing was implemented using the Illumina MiSeq platform at Genesky Biotechnologies Inc., (Shanghai, China). The details of soil DNA extraction and high-throughput sequencing are available in the Supplementary material. After quality control, the final total data set retained 3179 and 2264 operational taxonomic units (OTUs) numbers and 79875 and 79761 average clean reads for bacteria and fungi, respectively. Sequence Read Archive (SRA) database under accession number PRJNA1475409.

Soil gravimetric water content was measured by drying the samples at 105 °C to a constant weight. After the soil was air-dried, a total organic carbon analyzer (Vario TOC, Elementar, Germany) was used to measure soil total C, a Kjeldahl apparatus (Kjeltec 8,400, FOSS, Denmark) was used to determine soil total N, and an automatic discontinuous chemical analyzer (Smartchem 450, AMS, Italy) was used to measure soil total P. Soil pH was estimated by a water suspension (water/soil = 5:1) and a pH meter (pH S-3C, INESA, China).

### Statistical analyses

One-way ANOVA was used to test the effects of restoration year on aboveground biomass and soil properties across restoration statuses. Additionally, we test changes in bacterial and fungal alpha diversity across different restoration years using the one-way ANOVA. The Nonmetric Multidimensional Scaling (NMDS) is used to visualize the effects of different restoration years on the microbial beta diversity.

The co-occurrence network analysis was used to identify the intrinsic relationships within microbial communities and the correlations between microbial communities and plants. In detail, the co-occurrence network was analyzed at the species level (average abundance of more than 0.1% to ensure the accuracy of Spearman correlations) using the R program, and the network topological features were calculated with the “*igraph*” package (Lupatini et al., 2014; Jiang et al., 2018). A correlation analysis was conducted by calculating all possible Spearman rank correlations among the microbial species to visualize the associations in the network. According to the “*psych*” and “*multtest*” package, the coefficient ρ > 0.6 of Spearman’s statistical correlation was regarded as valid co-occurrence, and all *P*-values (*P* < 0.01) were adjusted by using the false discovery rate (FDR) for multiple testing (Benjamini et al., 2006). The nodes in the structured networks represent bacterial or fungal taxa (species level), and links represent significant correlations between taxa. These microbial taxa (species level) were obtained after excluding low-quality reads (quality score < 20 or sequences shorter than 100 bp) and clustering at a 97% identity cut-off using the UPARSE pipeline. Subsequently, the clusters with singleton sequences were removed. The taxonomies of unique operational taxonomic units (OTUs) were annotated using the RDP Classifier. We used Gephi (0.9.2) to construct and analyze the networks, and we conducted robustness analysis to assess the stability of the co-occurrence network. The co-occurrence network statistics include the number of nodes and links, the clustering coefficient, the average degree, the average path length, the modularity, and the graph density. Likewise, the random network (Erdös-Rényi network) with the same number of nodes and edges as the actual network was constructed using the subnet_property function of the “*meconetcomp*” package. These sub-networks (i.e., random network) were obtained by subsetting the global network dataset based on the OTUs present in each sample. Sub-networks retaining only the OTUs present in that sample and the edges among them, and then computing the network topological properties. These sub-network topological features across varying restoration statuses and years were used to analyze the effects of restoration years and environmental factors on the microbial co-occurrence network complexity. Moreover, we established a network of relations between microbial communities and different plant species. The method of relation network analysis was the same as co-occurrence (Spearman’s coefficient ρ > 0.6 and *P* < 0.01).

We used the “*randomForest*” and “*A3*” packages to structure the random forest regression model and analyze the importance of soil properties on microbial co-occurrence network complexity. Likewise, we conducted the correlation (Pearson) analyses with the soil properties against microbial co-occurrence network complexity. All statistical analyses were performed using R 4.0.2 (R Core Team, 2015).

## Results

### Plant traits and soil properties over the 5-year restoration period

In early restoration status, total plant biomass and *C. virgata* biomass gradually increased over the five restoration years, while *K. sieversiana* biomass gradually decreased. The moderate restoration status is characterized by *L. chinensis* + forbs, exhibiting high plant diversity, and *L. chinensis* is the dominant species. The vegetation type at late restoration status is *L. chinensis* + *L. daurica* + *Carex duriuscula*, with the biomass of *L. chinensis* significantly higher than in the early and moderate restoration statuses. *L. chinensis* has the highest biomass in a stable community status. During the five restoration years, the total plant biomass in moderate, late restoration, and stable community statuses did not increase significantly (Table 1).

Over the 5 years, soil total nitrogen (N) and total phosphorus (P) increased significantly during the early restoration status. In moderate restoration status, soil total P in 2023 was significantly higher than in other years, and soil pH in 2025 decreased significantly. In the stable community status, soil total P in 2025 was significantly lower than in other years. Soil water content was highest in 2025 and lowest in 2023 among the four statuses (Table 2).

**TABLE 2:** One-way ANOVA of the effects of different restoration years on soil properties in the varying restoration statuses (mean ± SE, *n* = 4).

Treatments		TC (mg kg^–1^)	TN (mg kg^–1^)	TP (mg kg^–1^)	pH	SW (%)
Early restoration status	2021	8307.50 ± 431.15 a	163.45 ± 9.68 b	199.05 ± 8.21 b	9.63 ± 0.20 a	7.97 ± 0.85 b
	2023	8910.00 ± 322.28 a	169.58 ± 10.89 b	221.18 ± 15.93 a	9.58 ± 0.15 a	6.35 ± 0.56 c
	2025	9027.50 ± 605.39 a	188.80 ± 4.31 a	231.58 ± 6.91 a	9.53 ± 0.21 a	9.29 ± 0.33 a
Moderate restoration status	2021	9725.00 ± 596.74 a	188.70 ± 15.78 a	204.25 ± 10.61 b	8.87 ± 0.13 a	7.11 ± 0.50 b
	2023	10375.00 ± 670.85 a	206.45 ± 11.44 a	252.05 ± 15.28 a	8.93 ± 0.14 a	6.26 ± 0.27 c
	2025	10202.50 ± 485.34 a	204.40 ± 13.59 a	222.78 ± 8.56 b	8.54 ± 0.08 b	9.06 ± 0.61 a
Late restoration status	2021	10630.00 ± 381.23 a	236.25 ± 13.70 a	245.80 ± 13.50 ab	8.23 ± 0.17 a	8.33 ± 0.43 b
	2023	10857.50 ± 1329.82 a	244.98 ± 12.73 a	276.25 ± 33.75 a	8.15 ± 0.07 a	6.77 ± 0.40 c
	2025	10955.00 ± 1215.17 a	236.55 ± 19.45 a	229.40 ± 4.89 b	8.02 ± 0.11 a	9.12 ± 0.19 a
Stable community status	2021	12730.00 ± 1798.37 a	245.15 ± 13.29 a	270.30 ± 13.10 a	8.03 ± 0.26 a	8.87 ± 0.40 b
	2023	13122.50 ± 1187.98 a	246.43 ± 12.45 a	275.03 ± 28.80 a	7.98 ± 0.12 a	7.16 ± 0.28 c
	2025	11987.50 ± 1705.90 a	253.40 ± 20.88 a	231.70 ± 3.86 b	7.97 ± 0.04 a	9.40 ± 0.27 a

Different letters indicate a significant difference (*P* < 0.05) among the 3 years. TC, soil total carbon; TN, soil total nitrogen; TP, soil total phosphorus; pH, soil pH; SW, soil water content.

### Soil microbial diversity and co-occurrence network

**FIGURE 1:**
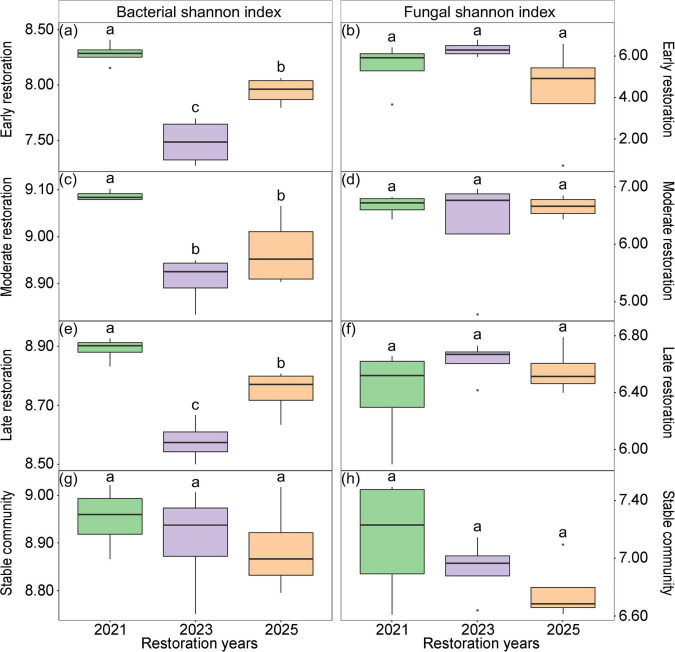
Bacterial **(a,c,e,g)** and fungal **(b,d,f,h)** Shannon index among the three restoration years. Vertical bars represent mean ± SE, *n* = 4. Different letters indicate a significant difference (*P* < 0.05) among the 3 years.

**FIGURE 2:**
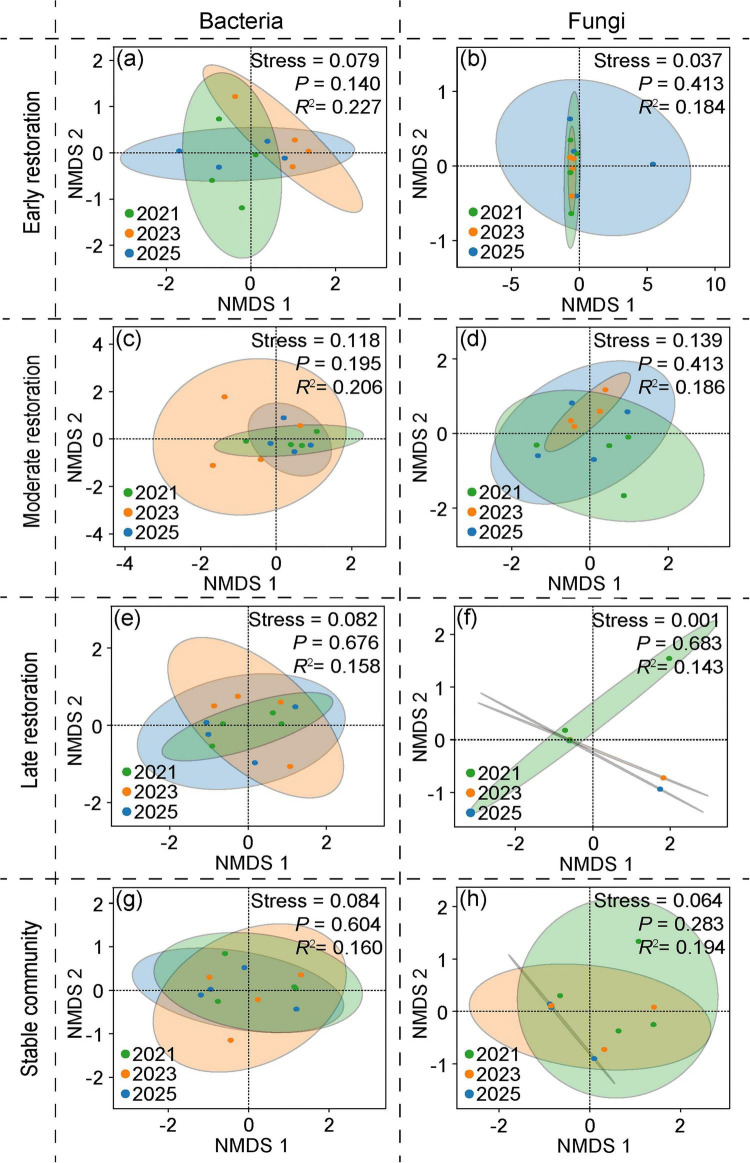
Nonmetric multidimensional scaling (NMDS) of soil bacterial **(a,c,e,g)** and fungal **(b,d,f,h)** community structure in the different restoration statuses. Different colors represents the variation of microbial community composition in different years based on Bray-Curtis distance (95% confidence intervals).

During the 5-year period, the bacterial Shannon index showed a fluctuating trend, initially decreasing and then increasing in most restoration statuses, whereas the fungal Shannon index did not change significantly among treatments (Figure 1). Moreover, nonmetric multidimensional scaling (NMDS) analysis showed that microbial β-diversity (bacteria and fungi) did not differ significantly among the 3 years (Figure 2).

**FIGURE 3:**
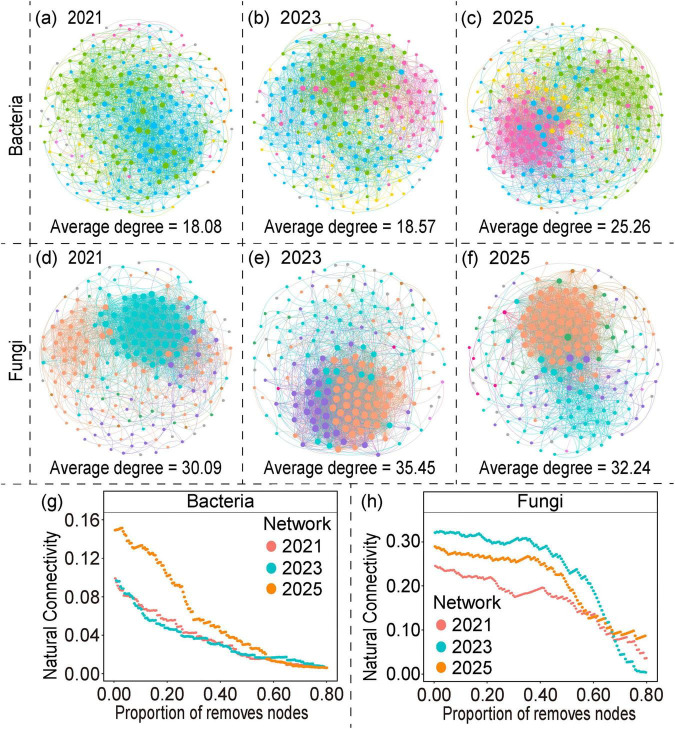
Co-occurrence network **(a–f)** and robustness analysis **(g,h)** of microbial communities based on the species level in different years. Each dot represents a bacterial phylotype (species level). Different colors represent distinct modules in the same co-occurrence networks, as determined using the modularity algorithm in Gephi with default settings. The nodes within a module imply potential functional similarity and niche overlap in the ecosystem. Distinct colors represent the niche partitioning of microbial taxa across the network and partially predict the associations between network topology and various soil functions. The connections stand for a strong (Spearman’s ρ > 0.6) and significant (*P* < 0.01) correlation. The node sizes reflect their degree of connectivity (the number of edges assigned to the node). Robustness analysis shows the relationship between the natural connectivity of microbial communities and the proportion of removed nodes, such that larger shifts for the same number of removed nodes indicate lower stability and robustness within microbial networks.

**FIGURE 4:**
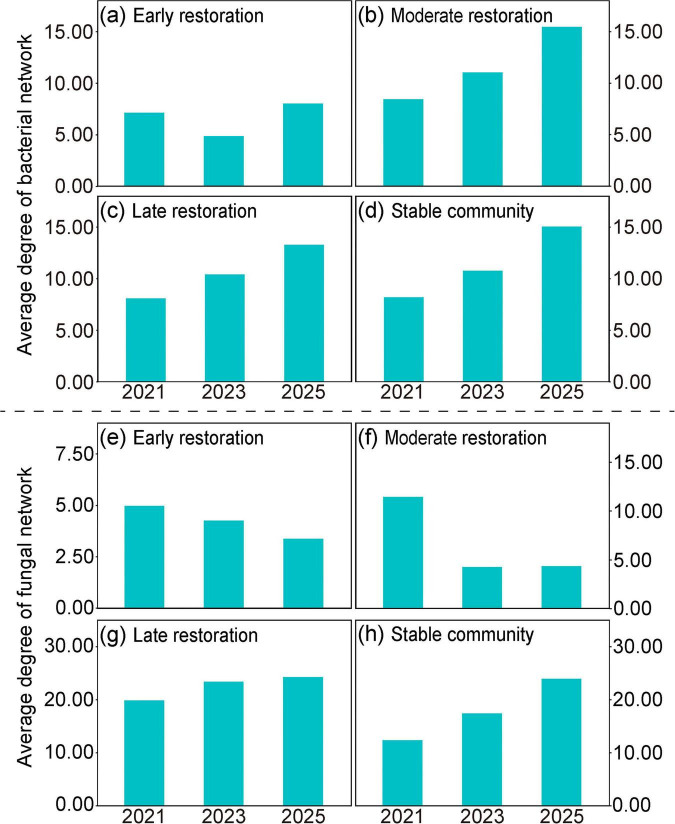
Bacterial **(a–d)** and fungal **(e–h)** sub-networks degree in different restoration statuses. Vertical bars represent the mean degree for each restoration status (*n* = 4). These sub-networks were generated from the bacterial and fungal co-occurrence network.

The bacterial network complexity and stability gradually increased over the five restoration years (Figures 3a–c,g). The fungal network complexity and stability showed a fluctuating trend (Figures 3d–f,h). Subsequently, the degree of sub-networks was used to represent the microbial network complexity in each sample. The bacterial network complexity in the early restoration status showed a fluctuating trend, and it gradually increased in the other restoration statuses (Figures 4a–d). The fungal network complexity in early and moderate restoration statuses gradually decreased, whereas it gradually increased in late restoration and stable community statuses (Figures 4e–h).

### The microbial network-plants interactions and affecting factors

**FIGURE 5:**
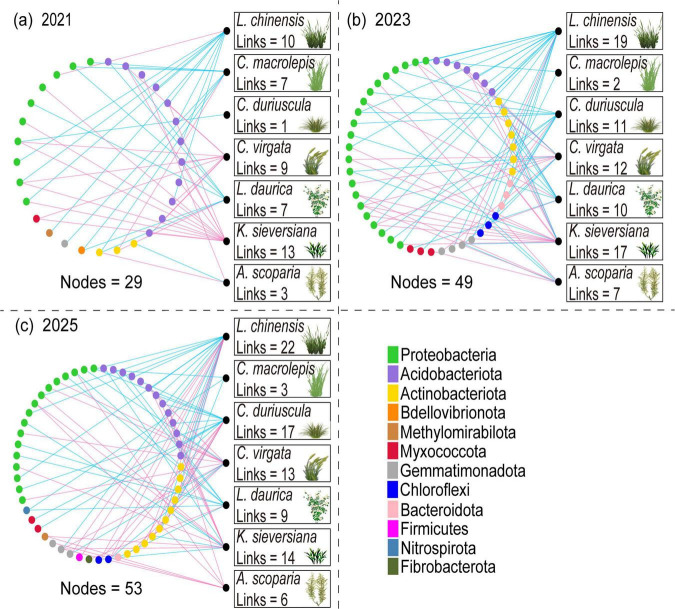
Relation analysis between soil bacterial network (species level) and plant biomass in different years. The blue and red lines represent positive and negative correlations (Spearman’s ρ < 0.6, *P* < 0.01) between microbial communities and plant biomass. The nodes are colored at the phylum level.

**FIGURE 6:**
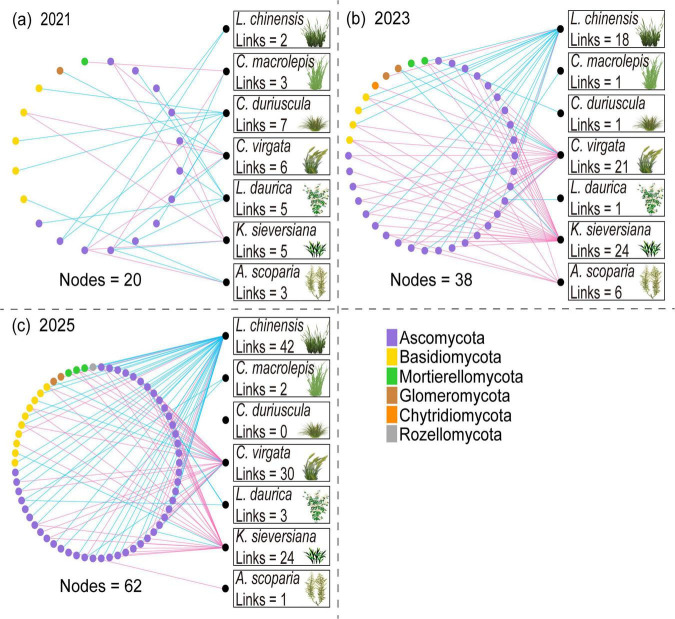
Relationships between soil fungal network (species level) and plant biomass in different years. The blue and red lines represent positive and negative correlations (Spearman’s ρ < 0.6, *P* < 0.01) between microbial communities and plant biomass. The nodes are colored at the phylum level.

The relation network analysis showed that plant-microbe interactions increased over the years of restoration. In detail, *L. chinensis*, *C. duriuscula*, and *L. daurica* showed positive correlations with the bacterial network, and these correlations increased gradually during the 5 years. *C. virgata*, *K. sieversiana*, and *A. scoparia* showed a negative correlation with the bacterial network (Figure 5). Over the five restoration years, the positive correlations with the fungal network gradually increased for *L. chinensis* but decreased for *C. duriuscula*. *C. virgata* and *K. sieversiana* had negative correlations with the fungal network (Figure 6).

**FIGURE 7:**
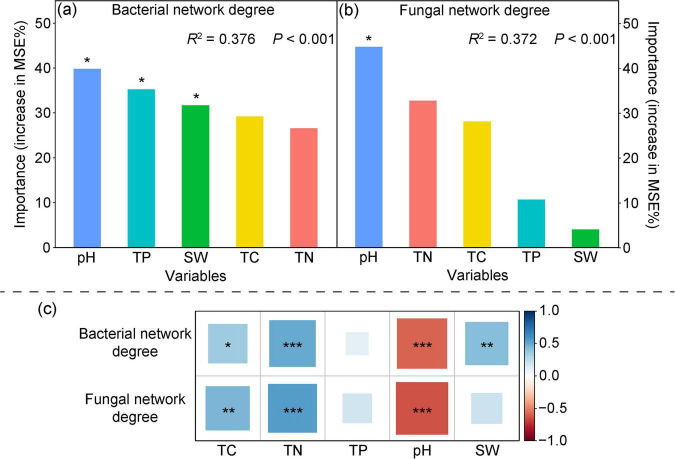
Random forest regression model **(a,b)** and Pearson correlation **(c)** indicate the main soil properties drivers of bacterial and fungal degree. The accuracy importance measure of the random forest regression model is computed for each tree and averaged over the forest (5000 trees), and determined by the increase in MSE (mean squared error). *P < 0.05 on the bar **(a,b)** shows that the associated soil properties have a significant effect on microbial degree. The blue and red squares **(c)** represent positive and negative correlations between soil properties and microbial degree. ***, *P* < 0.001; **, *P* < 0.01; *, *P* < 0.05; TC, total carbon; TN, total nitrogen; TP, total phosphorus; pH, soil pH; SW, soil water content.

Random forest regression model revealed that soil pH is the primary factor affecting bacterial and fungal networks (Figures 7a,b). Pearson correlation demonstrated that soil total carbon (C) and total N are positively correlated with microbial network complexity, whereas soil pH is negatively correlated with it. Furthermore, bacterial network complexity had a positive correlation with soil water content (Figure 7c).

## Discussion

### The restoration of plants and soil in the varying restoration statuses

Over the 5 years, a significant increase in biomass was observed only in the early restoration status, and the vegetation type shifted to a *C. virgata* community (Table 1). These results indicate that the early restoration status showed a recovery trend over the short-term period. However, this recovery does not imply that early restoration status has the potential to restore to the non-degradation status (Sun et al., 2020; Zhang et al., 2021). Soil properties and nutrient content remain the primary limiting factors in the early restoration status (Li et al., 2023). Changes in community type may be due to *C. virgata* having stronger competitive ability for light and soil nutrients than *K. sieversiana* (Zhou et al., 2019; Hautier et al., 2009; Li et al., 2020). Our results also indicated that vegetation in the moderate restoration status requires more time for restoration, which is consistent with a previous study (Liu M. et al., 2025). This phenomenon may be due to the slow rate of soil recovery, as soil in the moderate restoration status may take more than 10 years to recover (Bai et al., 2020). Additionally, our results suggested that the plant community in the late restoration status cannot be restored to the stable community status in the short term. The reason may be attributed to a strong soil heterogeneity in the Songnen grassland (Gao et al., 2019). This soil heterogeneity may lead to differences in soil nutrient content among the different areas of Songnen grassland (Liu et al., 2021). Plant communities in some areas may have more soil nutrient limitations. In grassland restoration, severe soil nutrient limitations may result in more challenging recovery of the soil environment, thus requiring a longer restoration period.

We found that soil total nitrogen (N) and phosphorus (P) gradually increase in the early restoration status and fluctuate in the moderate and late restoration statuses (Table 2). These results indicate that soil in the early restoration status may be undergoing recovery. Firstly, fencing enclosures promote plant growth and eliminate soil compaction, thereby increasing soil N content via alleviating soil denitrification (Dong et al., 2012; Liu et al., 2020). Secondly, increasing total plant biomass contributes to higher soil organic matter (Liu et al., 2016). These organic matter inputs are decomposed by microorganisms, potentially increasing soil total N and P content (Wallenstein and Burns, 2011). However, compared with the stable community status, the early restoration status still showed low soil nutrition content and high pH (Table 2). Therefore, soil recovery in the early restoration status still requires a longer period (Sun et al., 2020). In moderate and late restoration statuses, soil moisture may explain fluctuations in soil total P across years. Due to high salinity and alkalinity, soils in the Songnen grassland exhibit strong water retention (Zeng et al., 2018; Gao et al., 2019). Consequently, increased precipitation raises soil moisture, intensifies leaching, and reduces soil phosphorus content in wetter years (Mi et al., 2025).

### Changes in soil microbes over the five restoration years

The microbial α diversity results showed that bacterial α diversity is more sensitive than fungal α diversity over the 5-year restoration period (Figure 1). Our finding may be due to the differential effects of the soil environment on bacteria and fungi in degraded grassland. In detail, the high soil pH caused by grassland degradation can inhibit fungal activity but increase bacterial activity (Rousk et al., 2010), thereby enhancing the bacterial community in response to changes in the soil environment. During the restoration succession, bacterial abundance and diversity are generally correlated with environmental factors such as soil nutrients and moisture, whereas fungi are associated with plant community composition (Dong et al., 2021; Cuartero et al., 2024). Consequently, the stable fungal diversity across different restoration statuses may be attributed to the lack of significant changes in plant community composition. Meanwhile, variations in bacterial diversity may arise from soil properties, particularly the changes in soil moisture over the 5-year period (Liu B. et al., 2025). Furthermore, soil microbial β diversity showed no significant differences over the 5 years (Figure 2). This finding indicates that the microbial community structures across different degradation statuses remained stable during the 5-year restoration period (Guo et al., 2019). In non-degraded grasslands, a stable microbial community structure enhances positive interactions between dominant species and microbes (Hou et al., 2023), thereby promoting plant growth. During restoration succession, the stable microbial community suggests that the successional process may remain steady over the short term (Du et al., 2025). Microorganisms and plants might be accumulating restoration potential, but did not inducing a significant shift in successional stages.

The co-occurrence network suggested that bacterial richness may gradually increase and form complex community structures over the 5 years (Figure 3). The increasing microbial network complexity may indicate the potential for grassland recovery (Mi et al., 2026). However, the restoration of degraded grassland is slow, which may be due to the gradual reduction in soil pH and the accumulation of nutrients takes more than 10 years (Feng et al., 2010; Sun et al., 2020). Consequently, soil microbes are influenced by the slow restoration process and long-term factors from similar soil environments (Jiang et al., 2010), which may lead to the formation of stable community structures (Yan et al., 2025). Interestingly, the bacterial network complexity in the early restoration status exhibited fluctuations (Figure 4a). This result may be due to soil improvement caused by the growth of pioneer species (Jing et al., 2015). Soil N and P significantly increased over the 5 years, leading to continuous shifts in bacterial communities (Liu et al., 2020; Mi et al., 2025), thus reducing the complexity of the bacterial network. Moreover, the fungal network complexity gradually decreased in the early and moderate restoration statuses (Figures 4e,f). The decrease in fungal network complexity may be due to high soil pH and severe environmental conditions in the early and moderate restoration statuses, which can restrict fungal growth and richness (Rousk et al., 2010; Zhang et al., 2024). Notably, both bacterial and fungal network complexity increased in the late restoration status (Figures 4c,g), suggesting a recovery trend from the late restoration status toward the stable community status. However, transitions among different restoration statuses often entail changes in plant community composition and soil functions, which can lead to corresponding changes in microbial networks (Jayaramaiah et al., 2026; Song et al., 2025). Generally, microbial network complexity is highly correlated with plant diversity (Finck et al., 2026). In the Songnen Plain, plant diversity in the late restoration status is higher than that in the stable community status (Liu et al., 2021; Zhang et al., 2024), which may promote a more complex microbial network in the late restoration status. Therefore, the shift from late restoration status to stable community status is likely to involve fluctuations in microbial networks, whereas a 5-year restoration period remains insufficient to induce such a change.

### Key factor affecting microbial network and grassland restoration

The relation network showed an increasing correlation between *L. chinensis* and microbial communities over the 5 years (Figures 5, 6), indicating a positive effect on grassland recovery during the 5-year restoration period. In detail, *L. chinensis* has a strong tolerance to drought, high soil pH, and low soil fertility (Wang et al., 2004). These characteristics result in *L. chinensis* being the pioneer species in grassland restoration and a dominant species in local grasslands (Liu et al., 2021; Zeng et al., 2018). As such, the increasing positive relationships between *L. chinensis* and microbes suggest that *L. chinensis* is accumulating competitive advantages during the grassland restoration. However, the results for *L. chinensis* biomass and microbial network complexity also indicated that the moderate- and late-restoration grasslands maintained their restoration statuses. Furthermore, we found that most microbes negatively correlated with *C. virgata* and *K. scoparia* (Figures 5, 6). In severe environments, *C. virgata* and *K. scoparia* can grow rapidly and exhibit strong salty alkaline tolerance (Lin et al., 2015). These traits result in *C. virgata* and *K. scoparia* becoming dominant species in early restoration succession (Hautier et al., 2009; Gao et al., 2019). During the restoration succession process, annual vegetation is gradually replaced by perennial plants with higher nutrient utilization efficiency (Kardol et al., 2006). The positive interactions between perennial plants (e.g., *L. chinensis*) and microbes diminish the competitive advantages of *C. virgata* and *K. scoparia* (Li et al., 2024; Dangi et al., 2024), thus increasing their negative interactions with microbes.

We found that high pH is the primary factor limiting the microbial network complexity (Figure 7). The high soil pH in this area is due to the severe soil salinization of degraded grassland (Singh, 2015; Jiang et al., 2010). Firstly, the alkaline salts that cause soil salinization originate from climate change and soil parent material (Wang L. et al., 2009). In non-degraded grassland, these alkaline salts are dissolved by rainfall and leached into groundwater through the soil non-capillary pores (Wang and Ba, 2008). Secondly, increasing soil compaction destroys these non-capillary pores, leading to the continuous accumulation of salts at the surface and thus forming the high soil pH characteristic that typifies this area (Steffens et al., 2008). Finally, during grassland restoration, high soil pH can lead to soil nutrient imbalances (Jiao et al., 2016; Mitsuta et al., 2025). Soil structural deterioration affects the normal growth of microbes and plants (Wang Z. et al., 2009), thereby inhibiting the ecological recovery of grassland. However, our results showed decrease in soil pH only under moderate restoration status, which may be due to the slow regulatory effects of plants and microbes on soil pH (Sun et al., 2020; Liu M. et al., 2025). Therefore, these grasslands exhibit strong recovery potential but remain in restoration status throughout the 5-year restoration period. High soil pH may represent one of the key factors constraining restoration succession.

Additionally, the study area is characterized by severe soil salinization, with high soil pH being the most representative feature. Previous studies have indicated that salinization in this region primarily affects microbial communities through soil pH (Bai et al., 2023; Zhang et al., 2024). However, this study did not directly measure the effect of salinization on microbial networks, and the regulatory role of soil pH on these networks was inferred primarily from the random forest regression model. Therefore, it is necessary to conduct experimental tests of soil pH and salinization as restoration interventions. Otherwise, the core objective of this study was to investigate how microbial networks in the “restoration successional series” (i.e., early, moderate, late restoration statuses, and the stable *L. chinensis* community) changed over a 5-year recovery period. To this end, this study constructed microbial networks using data from the same year to elucidate the overall trend of network changes in the entire “restoration successional series” over the 5 years. Although the selection of sample plots in this study ensured the similarity of the external environment as much as possible, inevitable differences still existed among restoration statuses and sampling quadrats from different years. Accordingly, future studies will establish pot experiments to strictly control environmental factors and verify the effects of high soil pH and salinization on grassland restoration.

## Conclusion

Our study examined short-term grassland restoration across different restoration statuses using microbial community dynamics. We found that the bacterial network in early restoration status exhibited fluctuations over 5 years, and the dominant plant shifted from *K. sieversiana* to *C. virgata*. The increasing microbial network complexity in moderate and late restoration statuses indicated the grassland’s recovery potential, whereas these restoration statuses showed no trend toward progression to the next successional status over a 5-year period. Moreover, high soil pH might limit the bacterial network complexity and alleviate the restoration process. However, the natural regulation of soil pH by vegetation and microbes may take a long time. It is necessary to conduct an intervention experiment to verify the influence of soil pH amelioration on grassland restoration succession. Overall, our study provided evidence that microbial network structure reflects grassland ecological restoration. It emphasized that artificially adjusting soil pH may be a key management practice for promoting grassland restoration.

## Data Availability

The datasets presented in this study can be found in online repositories. The names of the repository/repositories and accession number(s) can be found in the article/Supplementary material.
