# Supplementary material for: Microbial network and its relationship with plants reflect the restoration of grasslands during a five-year period
Source: Front Microbiol. 2026 Jul 29;17:1902699. doi: 10.3389/fmicb.2026.1902699 (PMC13462077; doi:10.3389/fmicb.2026.1902699)
Supplement: Supplementary file 1 [file Table_1.docx]

**Table S1** Bacterial and fungal network properties among the different restoration years.

| Network metrics | Bacteria | | |  | Fungi | | |
| --- | --- | --- | --- | --- | --- | --- | --- |
|  | 2021 | 2023 | 2025 |  | 2021 | 2023 | 2025 |
| Number of nodes | 260 | 233 | 240 |  | 211 | 188 | 189 |
| Number of links | 2350 | 2163 | 3031 |  | 3174 | 3332 | 3047 |
| Clustering coefficient | 0.549 | 0.537 | 0.580 |  | 0.686 | 0.726 | 0.717 |
| Average degree | 18.077 | 18.567 | 25.258 |  | 30.085 | 35.447 | 32.243 |
| Eigenvector Centrality | 0.012 | 0.016 | 0.015 |  | 0.007 | 0.001 | 0.004 |
| Modularity | 2.039 | 0.909 | 0.932 |  | 0.490 | 0.202 | 0.221 |
| Graph density | 0.070 | 0.080 | 0.106 |  | 0.143 | 0.190 | 0.172 |

Note: Each nodes represent the bacterial or fungal taxa (species level), and the links represent the interactions between the two microbial taxa.

**Table S2** Relation network properties between the soil microbial communities and plants.

| Network metrics | | Bacteria | | |  | Fungi | | |
| --- | --- | --- | --- | --- | --- | --- | --- | --- |
|  |  | 2021 | 2023 | 2025 |  | 2021 | 2023 | 2025 |
|  | |  |  |  |  |  |  |  |
| Total microbial nodes | | 29 | 38 | 53 |  | 20 | 38 | 62 |
|  |  |  |  |  |  |  |  |  |
| Total number of links | Positive | 23 | 47 | 44 |  | 20 | 24 | 48 |
|  | Negative | 27 | 31 | 40 |  | 11 | 48 | 54 |
|  |  |  |  |  |  |  |  |  |
| *L. chinensis* links | Positive | 9 | 17 | 14 |  | 2 | 16 | 37 |
|  | Negative | 1 | 2 | 8 |  | 0 | 2 | 5 |
|  |  |  |  |  |  |  |  |  |
| *C. macrolepis* links | Positive | 6 | 2 | 3 |  | 1 | 1 | 2 |
|  | Negative | 1 | 0 | 0 |  | 2 | 0 | 0 |
|  |  |  |  |  |  |  |  |  |
| *C. duriuscula* links | Positive | 1 | 10 | 11 |  | 7 | 1 | 0 |
|  | Negative | 0 | 1 | 6 |  | 0 | 0 | 0 |
|  |  |  |  |  |  |  |  |  |
| *C. virgata* links | Positive | 0 | 4 | 3 |  | 2 | 4 | 4 |
|  | Negative | 9 | 8 | 10 |  | 4 | 17 | 26 |
|  |  |  |  |  |  |  |  |  |
| *L. daurica* links | Positive | 6 | 7 | 8 |  | 5 | 1 | 3 |
|  | Negative | 1 | 3 | 1 |  | 0 | 0 | 0 |
|  |  |  |  |  |  |  |  |  |
| *K. sieversiana* links | Positive | 0 | 4 | 5 |  | 1 | 1 | 2 |
|  | Negative | 13 | 13 | 9 |  | 4 | 23 | 22 |
|  |  |  |  |  |  |  |  |  |
| *A. scoparia* links | Positive | 1 | 3 | 0 |  | 2 | 0 | 0 |
|  | Negative | 2 | 4 | 6 |  | 1 | 6 | 1 |

Note: Each nodes represent the bacterial or fungal taxa (species level). The links represent the interactions between the microbial taxa (species level) and plant species.

**FIGURE S1** Bacterial (a, c,, and e) and fungal (b, d, and f) Shannon index among the four restoration statuses. Vertical bars represent mean ± SE, n = 4. Different letters indicate a significant difference (*P* < 0.05) among the four restoration statuses. EAR, early restoration status; MOD, moderate restoration status; LAT, late restoration status; STA, stable community statuses.

**FIGURE S2** Bacterial (a, c,, and e) and fungal (b, d, and f) sub-networks degree in different restoration years. Vertical bars represent mean degree for each degradation statuses (n = 4). These sub-networks were generated from bacterial and fungal co-occurrence network.

**Supplementary material for high-throughput sequencing and analyses**

The soil DNA was extracted from 0.25 g of each replicate soil sample using the MoBio Powersoil TM DNA Isolation Kits (MoBio Laboratories, Inc., USA) according to the manufacturer’s instructions. The extracted DNA was evaluated using 1% agarose gel, and DNA concentrations were measured using a NanoDrop 2000 to confirm the final DNA concentration of each sample. The F: 5′-ACTCCTACGGGAGGCAGCA-3′, R: 5′-GGACTACHVGGGTWTCTAAT-3′ was used to amplify the V3-V4 regions of the bacterial rRNA. The F: 5′-CTTGGTCATTTAGAGGAAGTAA-3′, R: 5′-GCTGCGTTCTTCATCGATGC -3′ was used to amplify the ITS1 primer pairs, regions of the fungal rRNA. To distinguish the target gene amplicons originating from the different samples, a unique 8-bp barcode was added to the 5′ end of both the forward and reverse primers. Amplicon paired-end sequencing was implemented using the Illumina MiSeq platform at Genesky Biotechnologies Inc. (Shanghai, China).

Sequencing data was analyzed according to acknowledged guidelines (Schöler et al., 2017; Vestergaard et al., 2017). Initially, raw data were merged with the lowest overlap of 10 bp using FLASH (Magoc and Salzberg 2011), and then the low-quality sequences (quality score < 20) or sequences shorter than 100 bp were discarded using Trim Galore. The primer sequences, barcodes, unresolved nucleotides, and adapter were removed from the sequences by using Mothur (Schloss et al., 2009), and the sequences with more than two mismatches to primers were eliminated from the sample. Chimeras were subsequently checked and removed using USEARCH (Edgar 2010). The remaining high-quality sequences were converted to FASTA format and concatenated into a single file. Finally, high-quality reads were clustered at a cut-off of 97% identity using the UPARSE pipeline (Edgar 2013), and the clusters with singleton sequences were removed. The taxonomies of unique operational taxonomic units (OTUs) were annotated using the RDP Classifier (Wang et al., 2007). To reduce the impact of sequencing depth variation among the different samples, each sample was then rarefied to the same sequence depth for downstream analyses.

**References**

Edgar, R. C. (2010). Search and clustering orders of magnitude faster than BLAST. Bioinformatics 26(19), 2460-2461. https://doi.org/10.1093/bioinformatics/btq461

Edgar, R. C. (2013). UPARSE: highly accurate OTU sequences from microbial amplicon reads. Nat Methods 10(10), 996-998. https://doi.org/10.1038/nmeth.2604

Magoc, T., Salzberg, S. L. (2011). FLASH: fast length adjustment of short reads to improve genome assemblies. Bioinformatics 27(21), 2957-2963. https://doi.org/10.1093/bioinformatics/btr507

Schloss, P. D., Westcott, S. L., Ryabin, T., Hall, J. R., Hartmann, M., Hollister, E. B., Lesniewski, R. A., Oakley, B. B., Parks, D. H., Robinson, C. J. (2009). Introducing mothur: open-source, platform-independent, community-supported software for describing and comparing microbial communities. Appl. Environ. Microbiol. 75(23), 7537-7541. https://doi.org/10.1128/AEM.01541-09

Schöler, A., Jacquiod, S., Vestergaard, G., Schulz, S., Schloter, M. (2017). Analysis of soil microbial communities based on amplicon sequencing of marker genes. Bio. Fert. Soils 53, 485-489. https://doi.org/10.1007/s00374-017-1205-1

Vestergaard, G., Schulz, S., Schöler, A., Schloter, M. (2017). Making big data smart-how to use metagenomics to understand soil quality. Bio. Fert. Soils 53, 479-484. https://doi.org/10.1007/s00374-017-1191-3

Wang, Q., Garrity, G. M., Tiedje, J. M., Cole, J. R. (2007). Naive Bayesian classifier for rapid assignment of rRNA sequences into the new bacterial taxonomy. Appl. Environ. Microbiol. 73, 5261-5267. https://doi.org/10.1128/AEM.00062-07
